# Correlation Between Baseline 18F-FDG PET/CT Findings and CD38- and CD138-Expressing Myeloma Cells in Bone Marrow and Clinical Parameters in Patients with Multiple Myeloma

**DOI:** 10.4274/tjh.2017.0372

**Published:** 2018-08-05

**Authors:** Arzu Cengiz, Hayri Üstün Arda, Firuzan Döğer, İrfan Yavaşoğlu, Yakup Yürekli, Ali Zahit Bolaman

**Affiliations:** 1 Adnan Menderes University Faculty of Medicine, Department of Nuclear Medicine, Aydın, Turkey; 2 Adnan Menderes University Faculty of Medicine, Department of Internal Medicine, Aydın, Turkey; 3 Adnan Menderes University Faculty of Medicine, Department of Pathology, Aydın, Turkey; 4 Adnan Menderes University Faculty of Medicine, Department of Hematology, Aydın, Turkey

**Keywords:** Multiple myeloma, CD38/CD138 antigen, Positron emission tomography/computed tomography, PET/CT

## Abstract

**Objective:**

The aim of this study was to evaluate the relation between the rate of fluorine-18 (18F) fludeoxyglucose (FDG) uptake and CD38 and CD138 expression in myeloma cells in bone marrow and other clinical parameters in patients with multiple myeloma (MM).

**Materials and Methods:**

Patients with the diagnosis of MM who underwent 18F-FDG positron emission tomography/computed tomography (PET/CT) for initial staging were evaluated retrospectively. We analyzed a total of 42 patients (43-83 years old, mean: 64.4±9.9). Hematological and biochemical tests including hemoglobin, hematocrit, C-reactive protein, β2-microglobulin, creatinine, albumin, calcium, lactate dehydrogenase, and erythrocyte sedimentation rate were recorded. In bone marrow samples, plasma cell ratio and CD38 and CD138 immunohistochemical staining were evaluated. On PET/CT images, mean standardized uptake values (SUV_mean_) of the right anterior and posterior iliac crest and right proximal femora were calculated. The correlations between the average SUV_mean_ of bone marrow and CD38- and CD138-expressing myeloma cells and other parameters were analyzed by Spearman’s correlation test. Values of p<0.05 were considered statistically significant.

**Results:**

Types of MM were IgG_K_ (45%), IgG_L_ (21%), IgA_K_ (7%), IgA_L_ (10%), and others (17%). Thirty-two (76%) patients were at stage III according to the Salmon-Durie staging system. There was a statistically significant positive correlation between bone marrow FDG uptake and percentage of plasma cells in bone marrow and CD38 and CD138 expression in plasma cells (r=0.403, r=0.339, and r=0.409) and β2-microglobulin and C-reactive protein levels (r=0.676, r=0.541). There was a negative correlation between bone marrow FDG uptake and hemoglobin and hematocrit values (r=-0.377 and r=-0.368). Other hematological parameters were not correlated with FDG uptake in bone marrow.

**Conclusion:**

Increased FDG uptake is correlated with the percentage of CD38 and CD138 expression in plasma cells in bone marrow. In addition to initial staging, 18F-FDG PET/CT is useful in treatment planning and prognostic evaluation in MM patients.

## Abstract (tr)

**Amaç:**

Bu çalışmanın amacı multipl myelom (MM) hastalarında kemik iliği florin-18 (18F) fluorodeoxyglucose (FDG) tutulumu ile plazma hücrelerinde CD38, CD138 ekspresyonu oranı ve diğer klinik parametreler arasındaki ilişkiyi değerlendirmektir.

**Gereç ve Yöntemler:**

MM tanısı almış, ilk evreleme amacıyla 18F-FDG positron emisyon tomografi/bilgisayarlı tomografi (PT/BT) yapılan hastalar geriye dönük olarak değerlendirildi. Toplam 42 hasta analiz edildi (43-83 yaş, ortalaması: 64,4±9,9). Hematolojik ve biyokimyasal testlerden hemoglobin, hematokrit, C reaktif protein, beta 2-mikroglobulin, kreatinin, albumin, kalsiyum, laktat dehidrogenaz, sedimentasyon düzeyleri kayıt altına alındı. Kemik iliğinde plazma hücre düzeyi ve immünhistokimya ile CD38 ve CD138 boyanma oranına bakıldı. 18F-FDG PET/BT görüntülerinde sağ proksimal femur, sağ ön ve arka ilyak krest ortalama standart tutulum oranı (SUV_ort._) kaydedildi. Kemik iliği SUV_ort._ ve myelom hücrelerinde CD38, CD138 ekspresyonu ve diğer klinik parametreler arasındaki korelasyon Spearman korelasyon testi ile analiz edildi. P değerleri <0,05 olduğunda anlamlı kabul edildi.

**Bulgular:**

Hastaların %45’ini IgG_K_, %21’ini IgG_L_, %7’sini IgA_K_, %10’unu IgA_L_ ve %17’sini diğer myeloma tipleri oluşturuyordu. Otuz iki hasta (%76) Salmon-Durie sınıflamasına göre evre 3 idi. Kemik iliği FDG tutulumu ile plazma hücre oranı ve CD38, CD138 ekspresyonu arasında (r=0,403, r=0,339 ve r=0,409) ve beta 2-mikroglobulin ve C reaktif protein düzeyi arasında (r=0,676, r=0,541) istatistiksel anlamlı pozitif korelasyon vardı. Kemik iliği FDG uptake ile hemoglobin ve hematokrit değerleri arasında negatif korelasyon bulundu (r=-0,377 ve r=-0,368). Diğer hematolojik paremetreler kemik iliği FDG tutulumu arasında bir korelasyon saptanmadı.

**Sonuç:**

Kemik iliğinde artmış FDG tutulumu, plazma hücre oranı ve CD38, CD38 ekspresyonu ile ilişkilidir. MM hastalarında 18F-FDG PET/BT, ilk evreleme yanında tedavi planlaması ve prognostik değerlendirmede de yararlıdır.

## Introduction

Multiple myeloma (MM) is a plasma cell neoplasm and the second most common hematologic neoplasm, accounting for 1% of all cancers and 13% of hematologic malignancies [1].

Positron emission tomography/computed tomography (PET/CT) with fluorine-18 (18F) fluorodeoxyglucose (FDG) is a whole-body imaging method that provides anatomical and metabolic information and is a useful technique for staging and therapy monitoring in patients with hematologic malignancies [2,3]. It is very useful in myeloma for detecting skeletal and extramedullary lesions with a sensitivity of approximately 80%-90% and a specificity of 80%-100% [4]. It can contribute to prognostic evaluation of MM patients. In a previous study, the authors showed that the number of focal lesions detected by PET/CT is a predictor of worse disease prognosis and death in these patients [5]. Recently, a guideline for imaging techniques in the management of MM patients stated that FDG PET/CT is an efficient imaging method for consecutive monitoring of disease burden of patients with nonsecretory myeloma, oligosecretory myeloma, and extramedullary disease. It was also reported that 18F-FDG PET/CT can define more lesions than plain X-rays in 40%-60% of cases and can be used for initial diagnosis of MM and assessment of suspected solitary plasmacytoma [6]. Some authors reported that elevated uptake of 18F-FDG by tumor cells is related to the metabolic activity of the tumor in MM [7,8,9].

CD38 is a membrane antigen expressed in the course of early B-cell growth. It is not expressed on mature activated B cells, while it is significantly re-expressed on plasma cells [10]. CD138 is transiently expressed by immature B-lymphocyte precursors, is lacking on mature circulating B cells, and is significantly expressed again after the differentiation to plasma cells [11]. Plasma cells in MM are positive for CD38 and CD138 compared to usual plasma cells [12]. The rate of infiltrating plasma cells expressing CD38 and CD138 in the bone marrow of MM patients may be related to disease activity.

Some laboratory parameters such as anemia, hypoalbuminemia, hypercalcemia, and high β2-microglobulin (β_2_M), C-reactive protein (CRP), creatinine (Cr), and lactate dehydrogenase (LDH) are also prognostically relevant in patients with MM [13,14,15,16,17].

In this study, we aimed to evaluate the relation between the 18F-FDG uptake of bone marrow and the expression of CD38 and CD138 in plasma cells and clinical parameters in patients with MM retrospectively.

## Materials and Methods

### Study Design and Patient Population

From March 2013 to December 2016, all patients with newly diagnosed MM who underwent 18F-FDG PET/CT for initial staging were evaluated retrospectively. A total of 42 patients (20 males, 22 females) between 43 and 83 years old (mean ± SD: 64.4±9.9 years) were included in the study. Demographic and clinical data of the patients are shown in Table 1.

**Figure 1:**
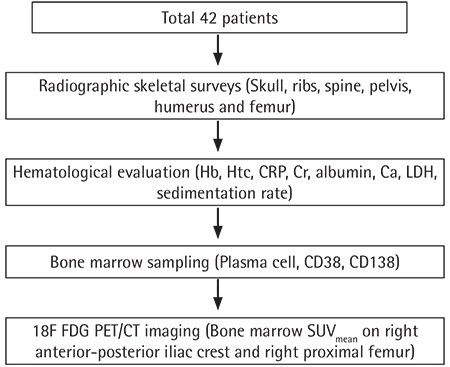
Flowchart of the study design. Hb: Hemoglobin, Htc: hematocrit, CRP: C-reactive protein; Cr: creatinine, Ca: calcium, LDH: lactate dehydrogenase.

The myeloma diagnosis was made based on the updated criteria of the International Myeloma Working Group [18]. Patients who were treated with chemotherapy, radiation therapy, or hematopoietic growth factor previously or who had a history of another malignancy or rheumatological disease were excluded. 18F-FDG PET/CT imaging was performed for all patients within 2 weeks of the initial diagnosis. Hematological and biochemical test results including levels of hemoglobin (Hb), hematocrit (Htc), CRP, Cr, albumin, calcium (Ca), LDH, and erythrocyte sedimentation rate were obtained for all patients within 7 days of PET/CT imaging. β_2_M levels were evaluated in fifteen patients. In the bone marrow specimens, the ratios of plasma cells to CD38 and CD138 immunohistochemical staining were evaluated. Conventional radiographic skeletal surveys of the skull, ribs, spine, pelvis, humerus, and femur were examined in all patients. The flowchart of the study design is shown in Figure 1.

The local ethics committee of Adnan Menderes University approved the study.

### 18F-FDG PET/CT Imaging

All patients’ fasting blood sugar levels were less than 180 mg/dL prior to imaging. After intravenous injection of 270-370 MBq of 18F-FDG, patients rested in a quiet room. Oral contrast was given to all patients. Whole-body imaging was performed after a resting period of 60 min using a Siemens Biograph mCT PET/CT scanner. The CT scan data were collected at 120 kV and 50 mAs. The PET acquisition was obtained from head to foot at a rate of 2 min/frame.

All FDG PET/CT images were evaluated visually and semiquantitatively by two nuclear medicine physicians. For semiquantitative evaluation, the mean standardized uptake value (SUV_mean_) of the right anterior and posterior iliac crests and the right proximal femur was calculated with a semiautomatic image registration software package. Femurs and iliac bones were chosen to standardize the calculation of bone marrow FDG uptake concordant with previous studies and bone marrow sampling [9,19]. The average SUV_mean_ was used for statistical analysis. To reduce the effect of an inhomogeneous distribution of tracer, SUV_mean_ was preferred to SUV_max_ for calculation of bone marrow FDG uptake.

### Immunohistochemical Staining

**Figure 2:**
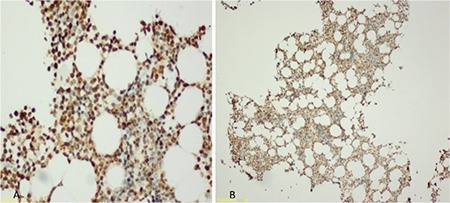
CD38 and CD138 expression detected by immunohistochemistry. A case with >90% CD38 positivity (A) and a case with >50% CD138 positivity (B).

Histopathological features in tissue preparations of patients with MM were evaluated. Plasma cell ratios in bone marrow were confirmed by Giemsa-stained aspirations. CD38 and CD138 immunohistochemical staining was applied (Santa Cruz Biotechnology, USA; Sc-7325, 200 µg/mL, 1/500 dilution). Immunohistochemical staining was done with an avidin-biotin complex system. All examinations were done with a light microscope (Olympus BX51, Japan). Cytoplasmic and membranous staining was taken into account. Staining was scored by counting at least 200 tumor cells in neighboring tumor areas where the staining was the most intense, and by measuring the ratio of stained cells to those not stained. Immunohistochemical staining in two cases is shown in Figures 2A and 2B.

### Statistical Analysis

Statistical assessment was done using SPSS 18.0 (SPSS Inc., Chicago, IL, USA). The correlations between semiquantitative values of bone marrow FDG uptake and CD38- and CD138-expressing myeloma cells and other clinical parameters were analyzed by Spearman’s rank correlation test. Values of p<0.05 were considered to be statistically significant.

## Results

Average SUV_mean _was between 0.73 and 13.84 (mean: 2.46±1.99). In bone marrow, the percentage of CD38-expressing myeloma cells ranged from 5% to 90% (mean: 38.9±22.82%) and that of CD138-expressing cells ranged from 5% to 90% (mean: 36.4±22.56%). There was a statistically significant positive correlation between bone marrow FDG uptake and CD38 and CD138 expression in plasma cells (p=0.030, r=0.339 and p=0.008, r=0.409). The ratio of plasma cells in bone marrow ranged from 5% to 80% (mean: 33.73±20.73%) and there was also a positive correlation between the ratio of plasma cells and FDG uptake of bone marrow (p=0.009, r=0.403). There was a negative correlation between SUV_mean _of bone marrow and Hb and Htc values (p=0.023, r=-0.377 and p=0.027, r=-0.368). Positive correlations between bone marrow FDG uptake and β_2_M (p=0.011, r=0.676) and CRP levels (p=0.001, r=0.541) were also detected. There were no correlations between bone marrow FDG uptake and albumin, Cr, Ca, sedimentation rate, or LDH levels. The mean hematological values and detailed statistical results are shown in Table 2.

## Discussion

18F-FDG PET/CT is an imaging procedure that can be used for initial evaluation of MM patients. This imaging method may aid to better specify osteolytic lesions, allowing for earlier detection of the disease. It can also define lesions in patients with negative results from conventional imaging methods. FDG PET/CT can define both medullary and extramedullary disease with reasonable success in one session in patients with MM, but this method may be suboptimal when there are diffuse bone marrow plasma cell infiltrations and lytic lesions in the skull [18]. 18F-FDG PET/CT can also be used for the estimation of prognosis in MM patients. Fonti et al. [5] reported that the numbers of focal lesions in 18F-FDG PET/CT or 99mTc-MIBI imaging and diffuse 99mTc-MIBI uptake are independent predictors for progression-free survival (PFS) and overall survival (OS) in patients with MM, but neither focal nor diffuse MRI pattern was an independent predictor of PFS or OS in a comparative study. They also concluded that 18F-FDG PET/CT or 99mTc-MIBI imaging must be performed at the time of initial diagnosis for specifying patients with worse outcomes who may be helped by more aggressive therapies [5].

CD38 is a type II transmembrane glycoprotein expressed on lymphoid and myeloid cells and also in nonhematopoietic tissues. It is highly expressed especially on MM cells. CD138 is a transmembrane heparan sulfate proteoglycan that provides some cellular functions including cell-cell adhesion and cell-matrix adhesion [10,11,12]. The presence of CD38 and CD138 revealed by immunohistochemical staining is a good indicator of plasma cells in bone marrow biopsy and CD38 and CD138 expressions have a diagnostic role in MM [20,21]. 

Anti-CD38 monoclonal antibodies such as daratumumab are important components of myeloma treatment [22,23,24]. Immunohistochemical studies have shown that CD138 is suitable for the identification and quantitation of normal and neoplastic plasma cells and thus helpful for the classification and assessment of malignant hematologic neoplasms. In addition, CD138 is an important marker in quantitation of the plasma cell population. Recently, anti-CD138 chimeric antigen receptor-modified T-cell treatment for MM has been reported [25].

There are only two studies related to PET/CT and plasma cell infiltration of bone marrow in MM patients. Ak and Gulbas [9] investigated 18F-FDG uptake and CD38/138 expression in the bone marrow of patients with MM. They reported that the FDG uptake of bone marrow was significantly related to the ratio of CD38/138-expressing plasma cell infiltration of bone marrow in patients with MM. In another study, Sager et al. [19] reported that there were significant correlations between bone marrow biopsy cellularity and plasma cell ratio and SUV_max_ values. The sensitivity of FDG PET in defining bone marrow involvement at initial diagnosis was 90% in this study.

In our study, we analyzed the association between FDG uptake of bone marrow and CD38- and CD138-expressing plasma cell infiltration ratio in bone marrow in patients with MM. Our study revealed that there was a statistically significant positive correlation between the percentage of CD38- and CD138-expressing plasma cells in bone marrow and FDG uptake of bone marrow (p=0.030 and p=0.008). This result suggests that increased FDG uptake of bone marrow is connected to the percentage of plasma cell infiltration of bone marrow in patients with MM. In addition, increased FDG uptake of bone marrow may be a marker for CD38 expression, which offers a possible therapeutic Ab target for the therapy of MM and thus may contribute to the selection of patients for immunotherapy. Additionally, after CD38 monoclonal antibody therapy, plasma cells that express CD38/138 are decreased. Thus, posttreatment FDG PET/CT imaging can also be used for estimation of monoclonal treatment. Further studies are required to validate the relationship between bone marrow FDG uptake and therapy with monoclonal antibodies.

In this study there was a negative correlation between SUV_mean_ of bone marrow and Hb and Htc rates (p=0.023, r=-0.377 and p=0.027, r=-0.368). It is known that FDG-18 uptake of bone marrow is increased in patients with anemia. It was also shown that hematopoietic growth factors may cause high FDG uptake of bone marrow [26]. To exclude the effect of these therapeutic agents, we included only pretreatment patients in this study.

In MM patients, some laboratory parameters including anemia, hypoalbuminemia, hypercalcemia, and elevated β_2_M, CRP, creatinine, and LDH are related to prognosis [13,14,15,16,17]. The correlation of pretreatment bone marrow FDG uptake with these prognostic factors may indicate a metabolic marker for poor prognosis in patients with MM. Park et al. [27] reported that SUV_max_ and number of hypermetabolic focal lesions on PET/CT images were positively correlated with prognostically relevant clinical factors. Ak and Gulbas [9] also showed a positive correlation between β_2_M and bone marrow FDG uptake values.

While there were positive correlations between bone marrow FDG uptake and β_2_M and CRP values, there were no correlations between FDG uptake value and albumin, Cr, Ca, sedimentation, or LDH values in our study. Although this study comprised a limited number of patients, these results showed that FDG-18 PET/CT may contribute to the identification of prognostically relevant clinical parameters, especially in the initial assessment of MM patients.

Our study has some limitations. Because it was designed as a retrospective study, we could not obtain the medical records of all patients. In addition, β_2_M levels were evaluated for only 15 of 42 patients. Due to incomplete data, we could not evaluate the relation between our results and the prognosis of the patients. Another limitation of the study was the relatively small number of cases.

## Conclusion

Increased FDG uptake is related to the percentage of plasma cell infiltration and CD38 and CD138 expression in plasma cells in the bone marrow. In addition to initial staging, 18F-FDG PET/CT is beneficial in therapy planning and prognostic assessment in patients with MM. Further studies with larger patient populations are required to validate the relation between bone marrow FDG uptake and CD38 and CD138 expression in plasma cells and other hematological parameters.

## Figures and Tables

**Table 1:**
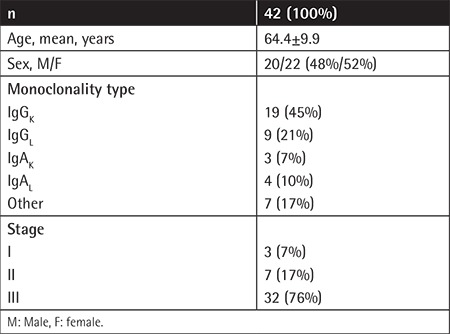
Demographic and clinical properties of patients.

**Table 2:**
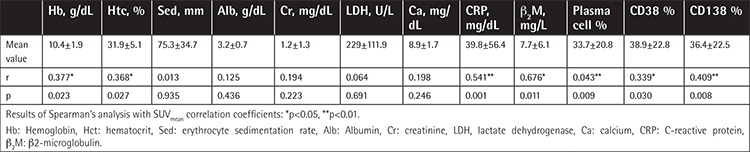
Hematological and biochemical parameters and plasma cell and CD38/CD138 ratios in patients.
